# The interconnection between cytokeratin and cell membrane-bound β-catenin in Sertoli cells derived from juvenile *Xenopus tropicalis* testes

**DOI:** 10.1242/bio.043950

**Published:** 2019-12-20

**Authors:** Thi Minh Xuan Nguyen, Marketa Vegrichtova, Tereza Tlapakova, Magdalena Krulova, Vladimir Krylov

**Affiliations:** 1 Charles University, Faculty of Science, Vinicna 7, 128 44, Prague 2, Czech Republic; 2 Department of Biotechnology, The University of Da-Nang, University of Science and Technology, 54 Nguyen Luong Bang, Da-Nang, 550000, Vietnam

**Keywords:** Cytokeratin, Adherens junctions, Immature Sertoli cells, *Xenopus tropicalis*, Testicles

## Abstract

Sertoli cells (SCs) play a central role in the determination of male sex during embryogenesis and spermatogenesis in adulthood. Failure in SC development is responsible for male sterility and testicular cancer. Before the onset of puberty, SCs are immature and differ considerably from mature cells in post-pubertal individuals regarding their morphology and biochemical activity. The major intermediate filament (IF) in mature SCs is vimentin, anchoring germ cells to the seminiferous epithelium. The collapse of vimentin has resulted in the disintegration of seminiferous epithelium and subsequent germ cell apoptosis. However, another IF, cytokeratin (CK) is observed only transiently in immature SCs in many species. Nevertheless, its function in SC differentiation is poorly understood. We examined the interconnection between CK and cell junctions using membrane β-catenin as a marker during testicular development in the *Xenopus tropicalis* model. Immunohistochemistry on juvenile (5 months old) testes revealed co-expression of CK, membrane β-catenin and E-cadherin. Adult (3-year-old males) samples confirmed only E-cadherin expression; CK and β-catenin were lost. To study the interconnection between CK and β-catenin-based cell junctions, the culture of immature SCs (here called XtiSCs) was employed. Suppression of CK by acrylamide in XtiSCs led to breakdown of membrane-bound β-catenin but not F-actin and β-tubulin or cell-adhesion proteins (focal adhesion kinase and integrin β1). In contrast to the obvious dependence of membrane β-catenin on CK stability, the detachment of β-catenin from the plasma membrane via uncoupling of cadherins by Ca2+ chelator EGTA had no effect on CK integrity. Interestingly, CHIR99021, a GSK3 inhibitor, also suppressed the CK network, resulting in the inhibition of XtiSCs cell-to-cell contacts and testicular development in juvenile frogs. This study suggests a novel role of CK in the retention of β-catenin-based junctions in immature SCs, and thus provides structural support for seminiferous tubule formation and germ cell development.

## Abstract (precis)

**Summary:** Cytokeratin (CK) and β-catenin are expressed in juvenile testicles and cultivated *Xenopus tropicalis* immature Sertoli cells (SC). Acrylamide and CHIR99021 disrupted the CK network, immature SC connections and testes development.

## INTRODUCTION

Sertoli cells (SCs) are located within seminiferous tubules and play a crucial role in male reproduction. In early development, anti-Müllerian hormone (AMH) is secreted by SCs to regress the female Müllerian tract, contributing to the determination of somatic male sex (Appert et al., 1998; Barrionuevo et al., 2011; Lee and Taketo, 1994). Later on, SCs provide nutrients, differentiation factors, appropriate mitogens to nourish the developing germ cells as well as regulate their maturation in adult testes (de Winter, 1993; Handelsman et al., 1990). Moreover, blood–testis barrier and immunosuppressive molecules produced by SCs protect sperm cells from attacks of immune system and establish the niche for the maturation of spermatogonial stem cells (SSCs) (Mital et al., 2010; Setchell, 2009). Failure in SC differentiation results in a male infertility due to a reduced production of sperm cells (Myers et al., 2005; Nistal et al., 1982).

Prior to puberty, SCs are maintained in an immature state with extensively different morphology and biochemical activity than mature cells. In contrast to constant cell division of immature SCs, the proliferative activity of the mature cells gradually decline to zero when tight junctions among adjacent SCs are established (Steinberger and Steinberger, 1971). At this point, fully differentiated SCs start to produce fluid into the lumen of seminiferous tubules leading to their expansion and subsequent enlargement of testis. The mature SCs also alter their protein expression, suppressing cytokeratin (CK) and AMH (Sharpe et al., 2003) and starting to produce inflammatory cytokine interleukin-1α (Wahab-Wahlgren et al., 2000) and androgen-binding protein to initiate spermatogenesis. The study of differences between immature and mature SCs is important for understanding SC differentiation and functional regulation.

CKs are the most abundant intermediate filaments (IF) in epithelial cells and the expression of their subsets is highly organ- or tissue-specific. In the cytoplasm, they form a complex network in association with membrane proteins implying their importance in some aspects of cell morphology and signaling. The transient expression of CK pair 8/18 (Stosiek et al., 1990) during SC differentiation is described in many species. CK is detected in the basal part of SCs from 12.5 days post coitum (dpc) in rat testes with well-differentiated seminiferous cords through to the early postnatal period, however, it is not found in mature cells (Paranko et al., 1986). In human, CK expression persists only in SCs of adult cryptorchid testes or peritumour tubules (Rogatsch et al., 1996). We performed the examination of CK expression in SCs of juvenile and adult *Xenopus* *tropicalis* testes. In agreement with the mammalian model, only SCs with Sox9 expression were positive for CK staining (Nguyen et al., 2019). However, even though CK has been considered as a marker of immature SCs (Rogatsch et al., 1996), its role in SC development is still poorly understood.

Cell junctions among SCs and between SCs and germ cells are crucial for testicular structure and spermatogenesis. In adulthood, the blood–testis barrier (BTB), formed by tight junctions among adjacent mature SCs, bisects seminiferous tubules into basal and apical compartments. Spermatogonia from the basal part differentiate and migrate, crossing the dynamic BTB to the apical compartment, which provides the microenvironment for the final differentiation of haploid cells into sperms and prevents spermatids from entering the blood and lymphs (Wen et al., 2016). Throughout this process, the immature germ cells are in tight contact with SCs (Wolski et al., 2005). In contrast to adulthood, spermatogonia of immature males rest and lay on the basal membrane of seminiferous tubules. This may require the specific setup of cell junctions and structure of developing SCs to anchor germ cells to the basement membrane and to protect them against the bloodstream. However, the cell-to-cell contacts in immature seminiferous cords have not yet been studied.

In the present study, we observed the co-expression of CK and β-catenin in SC junctions regarding seminiferous tubules of young testes. *Xenopus tropicalis* immature SCs (XtiSCs) have been established and described in a previous report (Tlapakova et al., 2016). *In vitro* treatment of XtiSCs with acrylamide and CHIR99021 showed the potent role of CK in the maintenance of β-catenin-based junctions. The disruption of CK in SCs of developing testis resulted in the failure in testicular maturation.

## RESULTS

### The role of CK in the retention of adherens junctions in immature SCs

**Fig. 1.**
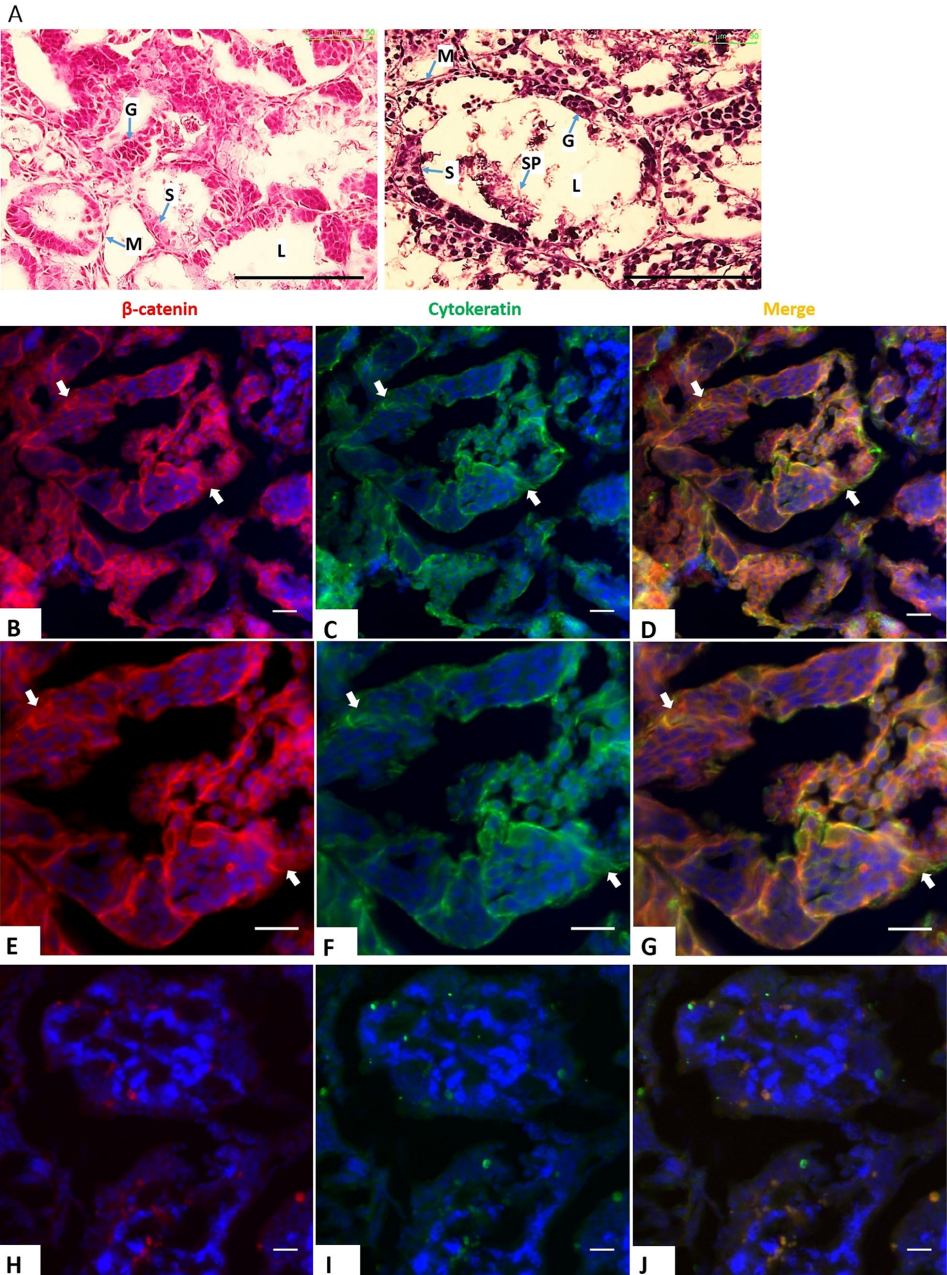
**Co-expression of CK and β-catenin during testicular development.** (A) H&E staining of 5-month-old (left) and 3-year-old (right) *X. tropicalis* testes. Scale bars: 100 μm. L, lumen; S, Sertoli cells; G, germ cells; SP, spermatid; M, mesenchyme. (B–J) Double staining of testicular sections of juvenile (B–G) and adult frogs (H–J) with CK (mouse, green) and β-catenin (rabbit, red) antibodies; E–G show higher magnification of young testes' staining. Nuclei were stained with DAPI (blue). Scale bars: 20 μm. Both proteins were found in the immature testes, but not in the adulthood. Arrows indicate SCs.

**Fig. 2.**
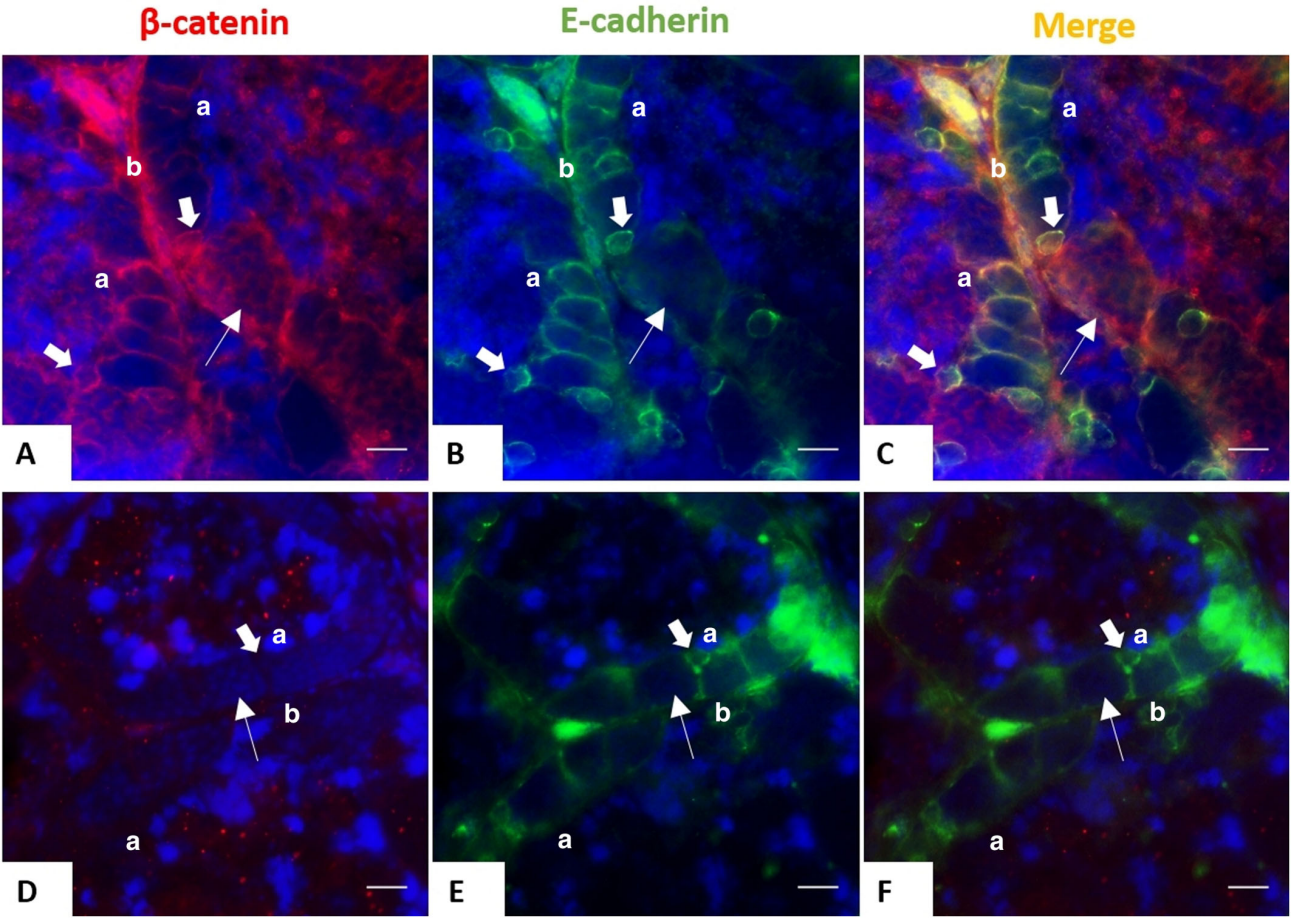
**Expression of E-cadherin and β-catenin in SCs during testicular development.** Double staining on testicular sections of juvenile (5-month-old) (A–C) and adult (3-year-old) frogs (D–F) with E-cadherin (mouse, green) and β-catenin (rabbit, red) antibodies. Nuclei were stained with DAPI (blue). Scale bars: 20 μm. Both proteins surrounded the SCs from juvenile testes, but only E-cadherin is expressed in adulthood. Thick arrows indicate SCs. Thinner arrows show germ cells. a, apical; b, basal.

β-catenin, a 92 kDa protein, is commonly found coupled with cell-to-cell junctions (Hartsock and Nelson, 2008) and has a high affinity for a transmembrane glycoprotein, E-cadherin (Huber and Weis, 2001). We examined the expression of CK, β-catenin and E-cadherin in juvenile (5-month-old) and adult (3-year-old) *X. tropicalis* testes. Hematoxylin and Eosin (H&E) staining of paraffin sections from juvenile testes showed well-organized seminiferous tubules, however they were small and narrow due to the incomplete development of tubular lumen compared to testes of 3-year-old individuals (Fig. 1A). Interestingly, immunostaining of CK and β-catenin revealed that only juvenile testes exhibited the expression of both proteins in SCs (Fig. 1B–J). Co-expression of β-catenin and E-cadherin surrounding SCs from young testes (Fig. 2A–C) implies the association of β-catenin with adherens junctions in immature SCs. Notably, β-catenin was also detected in germ cells of juvenile testes, but without positive CK and E-cadherin staining (Figs 1 and 2). The downregulation of β-catenin in mature testes indicates the change in cell-to-cell junctions of fully functional SCs to provide a structural support for spermatogenesis, even though E-cadherin is still present in the membrane of mature cells (Fig. 2D–F).

**Fig. 3.**
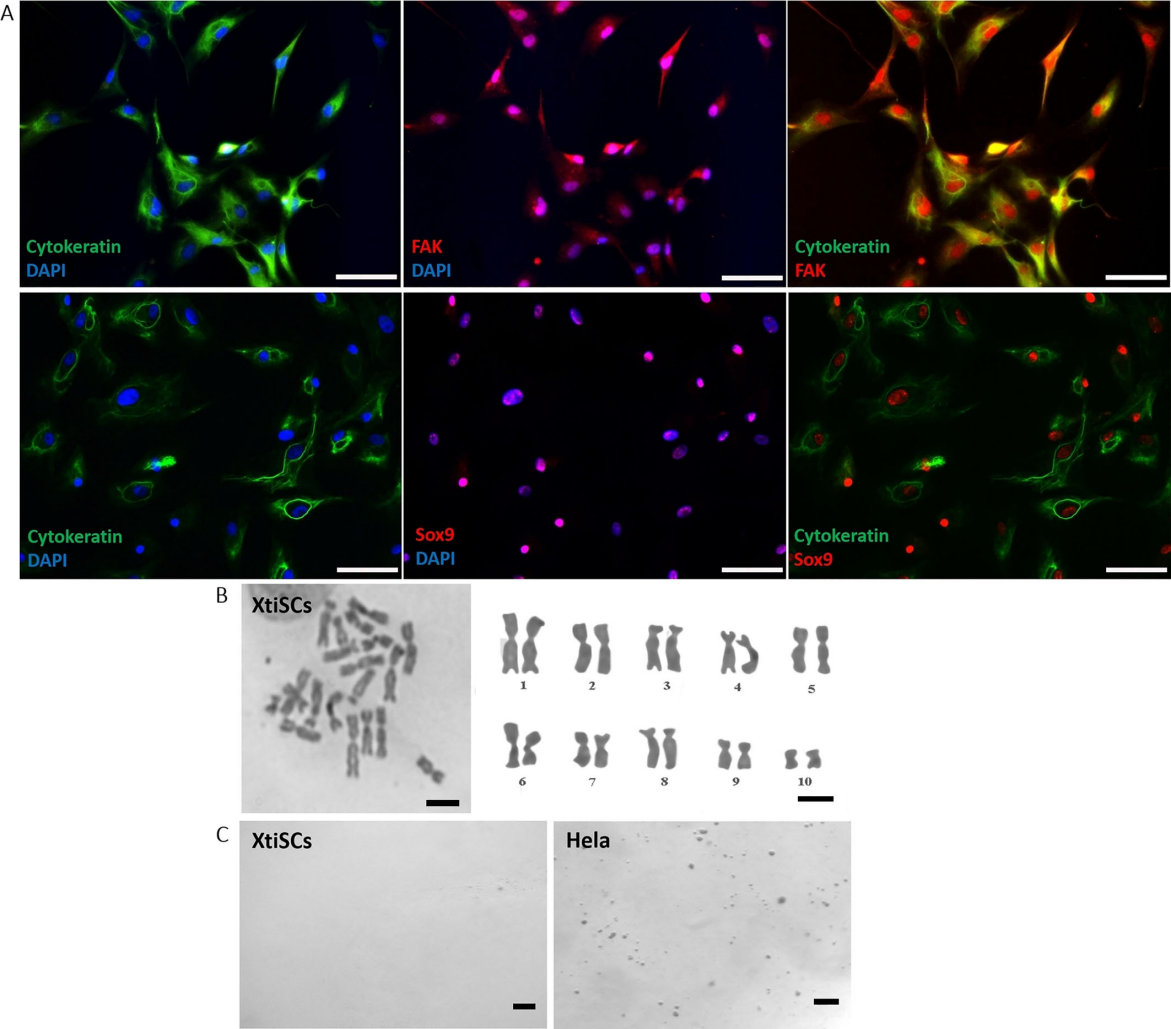
**Immunofluorescent, cytogenetic and transformation** **characteristics of isolated XtiSCs.** (A) XtiSCs expressed SC proteins, including focal adhesion kinase (FAK, red), Sox9 (red) and CK (green), an immature SC marker. Nuclei were stained with DAPI. Chromosome analysis (B) and soft agar assay (C) showed XtiSCs as non-transformed cells. Scale bars: 50 μm (A), 10 μm (B), 400 μm (C).

To reveal the role of CK in SC development we employed *in vitro* culture of immature SCs, called XtiSCs, previously isolated and described in Tlapakova et al. (2016). Cells expressed SC proteins, including Sox9, (Tlapakova et al., 2016), focal adhesion kinase (Fak) and CK (Fig. 3A). Chromosomal analysis showed normal karyotype even after 12 passages (Fig. 3B). In addition, XtiSC did not form colonies in soft agar, a typical feature of transformed cells (Fig. 3C).

**Fig. 4.**
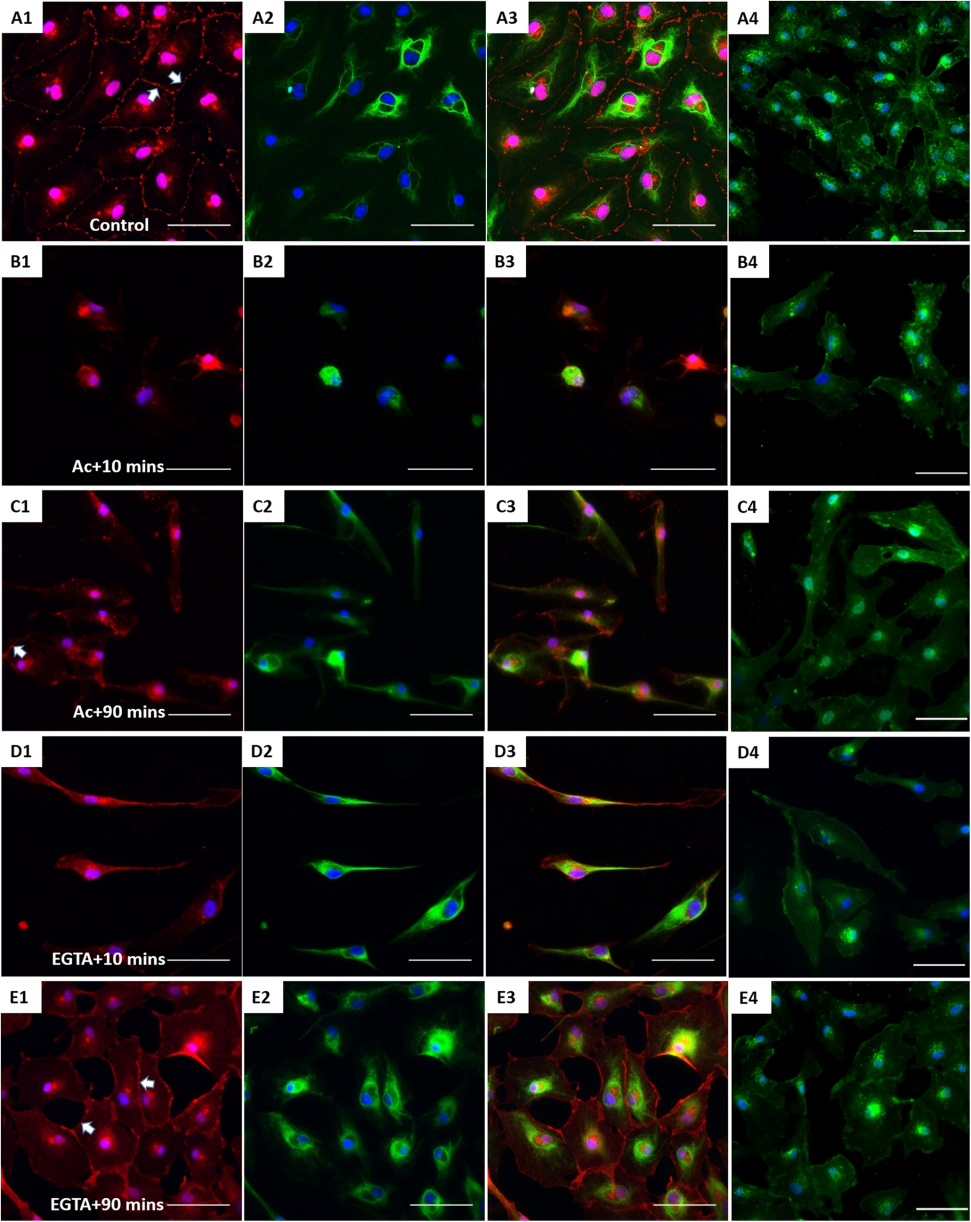
**The effect of CK network on the β-catenin-based cell junctions.** XtiSCs were treated with vehicles (Control, A) or 10 mM acrylamide (Ac; B,C) or 2 mM EGTA (EGTA; D,E). After treatment, cells were washed and changed to the fresh medium and then collected at the indicated time points: 10 min (Ac+10 min or EGTA+10 min, B,D) or 90 min (Ac+90 min or EGTA+90 min, C,E) for immunofluorescent staining with antibodies against β-catenin (red, A1–E1), CK (green, A2–E2) and merge (A3–E3). (A4–E4) Fluorescent images of WGA-stained cells showing the cell shape and cytoplasmic membrane. Arrows show membrane β-catenin. Nuclei were stained with DAPI (blue). Scale bars: 50 μm.

**Fig. 5.**
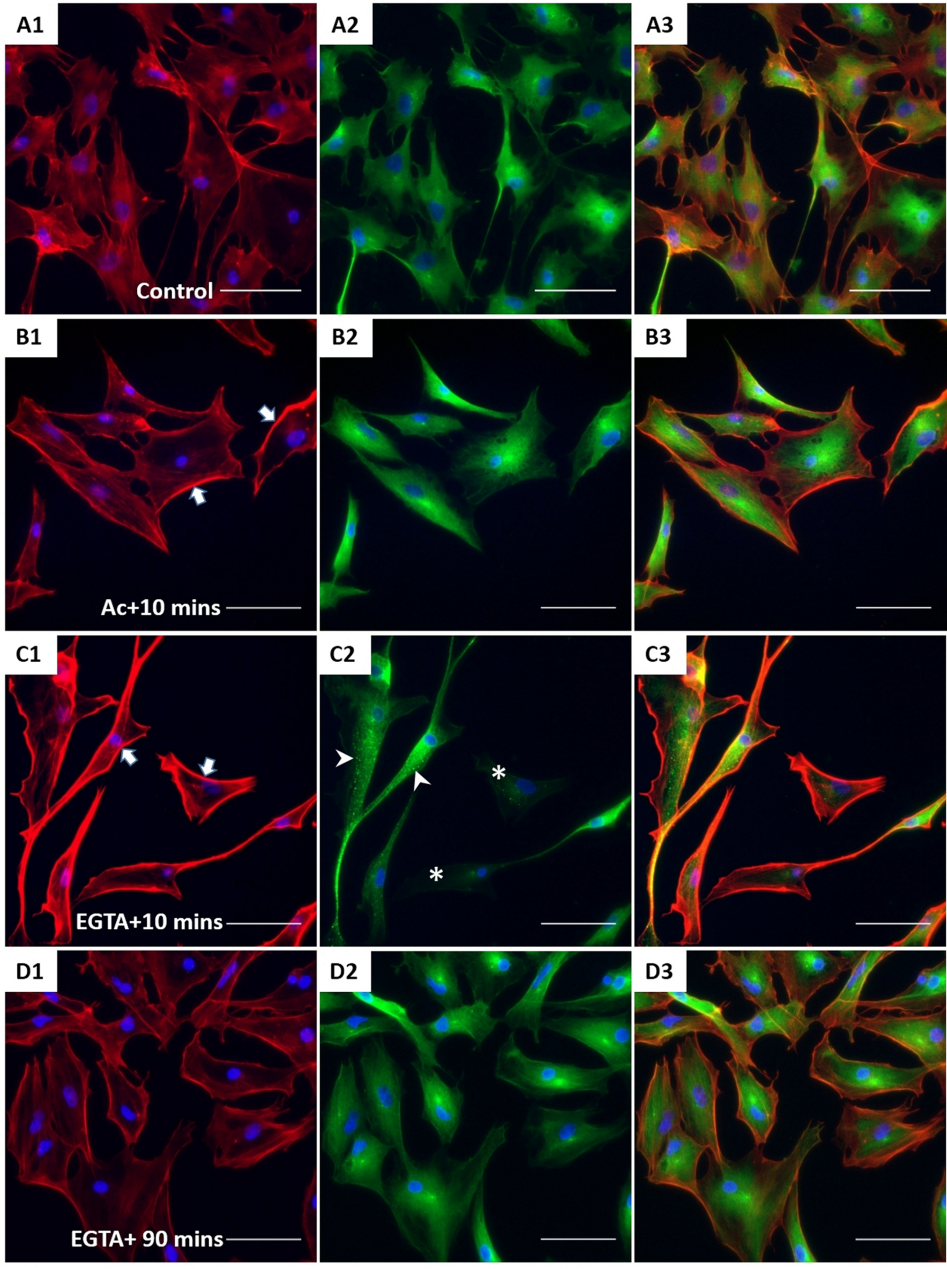
**The effect of acrylamide and EGTA on F-actin and tubulin.** After treatment, XtiSCs were collected at the indicated time points for immunofluorescent staining with antibodies against F-actin (red, A1–D1), β-tubulin (green, A2–D2) and merge (A3–D3). Arrows show thick membrane F-actin; the aggregates of β-tubulin are marked by arrowheads and asterisks indicate the cells without β-tubulin. Nuclei were stained with DAPI (blue). Scale bars: 50 μm.

**Fig. 6.**
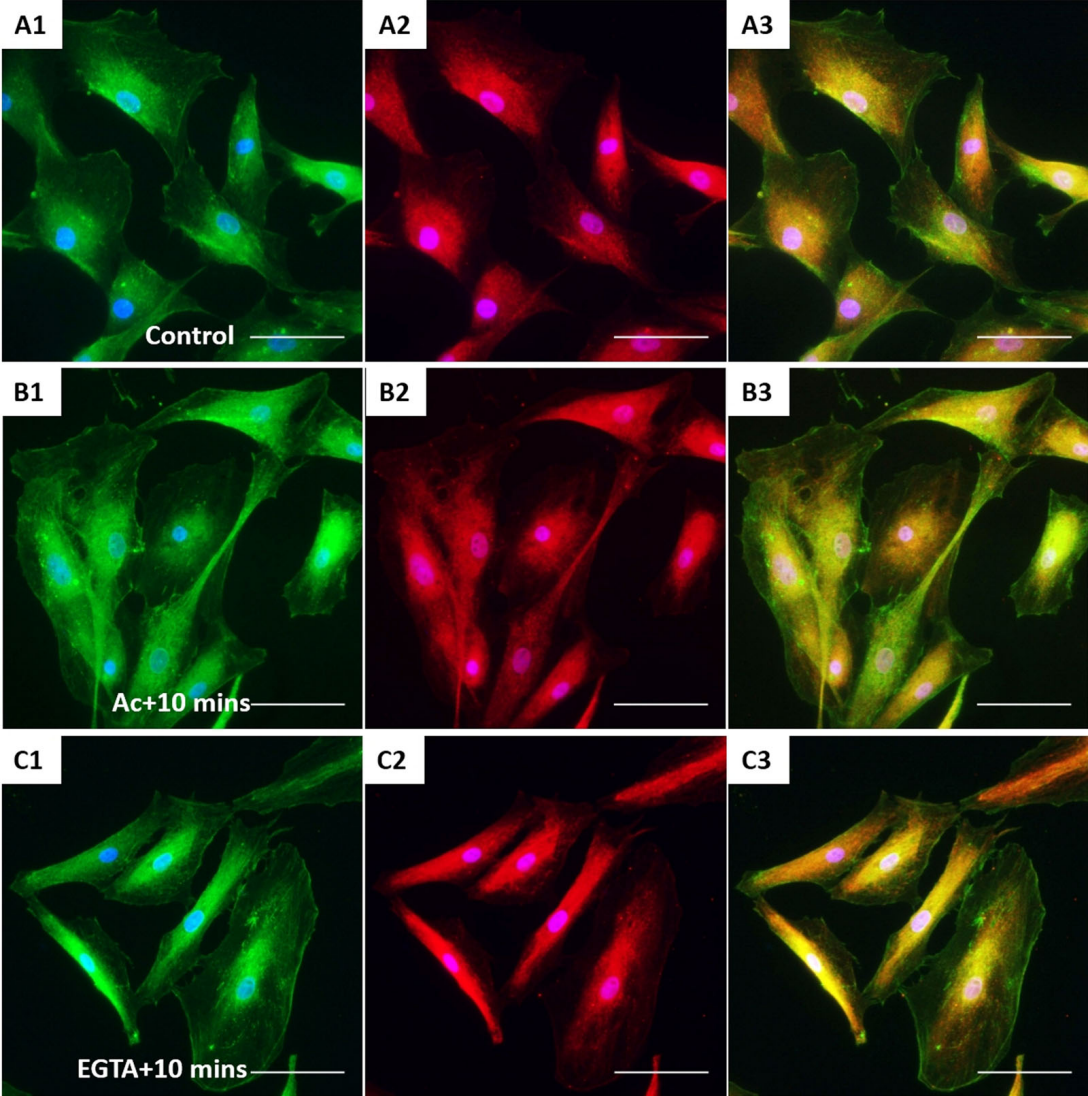
**The effect of acrylamide and EGTA on cell adhesion proteins.** Immunofluorescent images of control XtiSCs (A) and XtiSCs 10 min after washing out the acrylamide (B) or EGTA (C) staining with antibodies against integrin β1 (CD29, green, A1–C1), focal adhesion kinase (FAK, red, A2–C2) and merge (A3–C3). Nuclei were stained with DAPI (blue). Scale bars: 50 μm.

XtiSCs revealed strong staining with β-catenin antibody in both nuclei and cell membrane (Fig. 4A). β-catenin has dual biological functions, serving as a component of adherens junctions on the plasma membrane and as a transcription factor in cell nuclei (Daugherty and Gottardi, 2007). Firstly, we induced the disorganization of CK in XtiSCs to investigate its regulatory effect on membrane-bound β-catenin. The transient negative impact of acrylamide (unpolymerized form) on CK integrity has been well-described in *Xenopus* and mammals (Duncan et al., 2009; Shabana et al., 1994; Sonavane et al., 2017). The serious disruption of CK was also observed in XtiSCs after 75 min incubation with 10 mM acrylamide (Fig. 4B1), leading to the breakdown of β-catenin on the cell membrane (Fig. 4B2). Gradual recovery of membrane β-catenin coincidentally with the CK re-organization was obtained 90 min after the acrylamide was washed out from the culture medium (Fig. 4C). To examine the reverse process, i.e the effect of the downregulation of a membrane β-catenin on the stability of CK, we added a Ca2+ chelator EGTA to XtiSC culture to disrupt adherens junctions (cadherins) and subsequently destabilize β-catenin on the plasma membrane. 15 min treatment with 2 mM EGTA caused the disappearance of β-catenin from the cell membrane, and changed XtiSCs morphology into a fibroblast-like cell shape (Fig. 4D). However, EGTA didn't affect CK integrity (Fig. 4D2,E), resulting in full recovery of β-catenin on the cell membrane 90 min after washing out EGTA. The effect of acrylamide and EGTA on other cytoskeletal and cell adhesion proteins was also examined. Cortical actin filaments became predominant to probably enhance the recruitment of β-catenin-based cell-to-cell adhesions disrupted by both drugs (Fig. 5B1,C1) (Engl et al., 2014; Wu et al., 2014). While β-tubulin was not affected by acrylamide (Fig. 5B2), it formed aggregates after the treatment with 2 mM EGTA (Fig. 5C2). No changes in cell–matrix adhesion molecules (FAK and integrin β1) were observed (Fig. 6). Taken together, these results suggest that the CK network is required to sustain the β-catenin-based cell junctions in XtiSCs, whereas CK expression has no substantial effect on the stability of membrane β-catenin.

GSK3-mediated phosphorylation is responsible for β-catenin degradation by the ubiquitin–proteasome pathway. Interestingly, in the previous study, we observed the suppression of CK in XtiSCs by CHIR99021, a GSK-3 inhibitor (Nguyen et al., 2019). Thus, to determine the relationship between CK expression and β-catenin we added CHIR99021 or lithium chloride (LiCl), another GSK-3 inhibitor (Kramer et al., 2012), to XtiSC culture. Reactivation of cytosolic GSK-3 was achieved by the IWP2, an inhibitor of Wnt secretion (García-Reyes et al., 2018), which subsequently releases GSK-3 from the blocking complex, LRP-associated Wnt ‘signalosome’ (Bilic et al., 2007). XtiSCs did not respond to 10 mM LiCl after a 3-day treatment. In 20 mM and 40 mM supplemented media, cells died after 24 h and 6 h, respectively (data not shown).

**Fig. 7.**
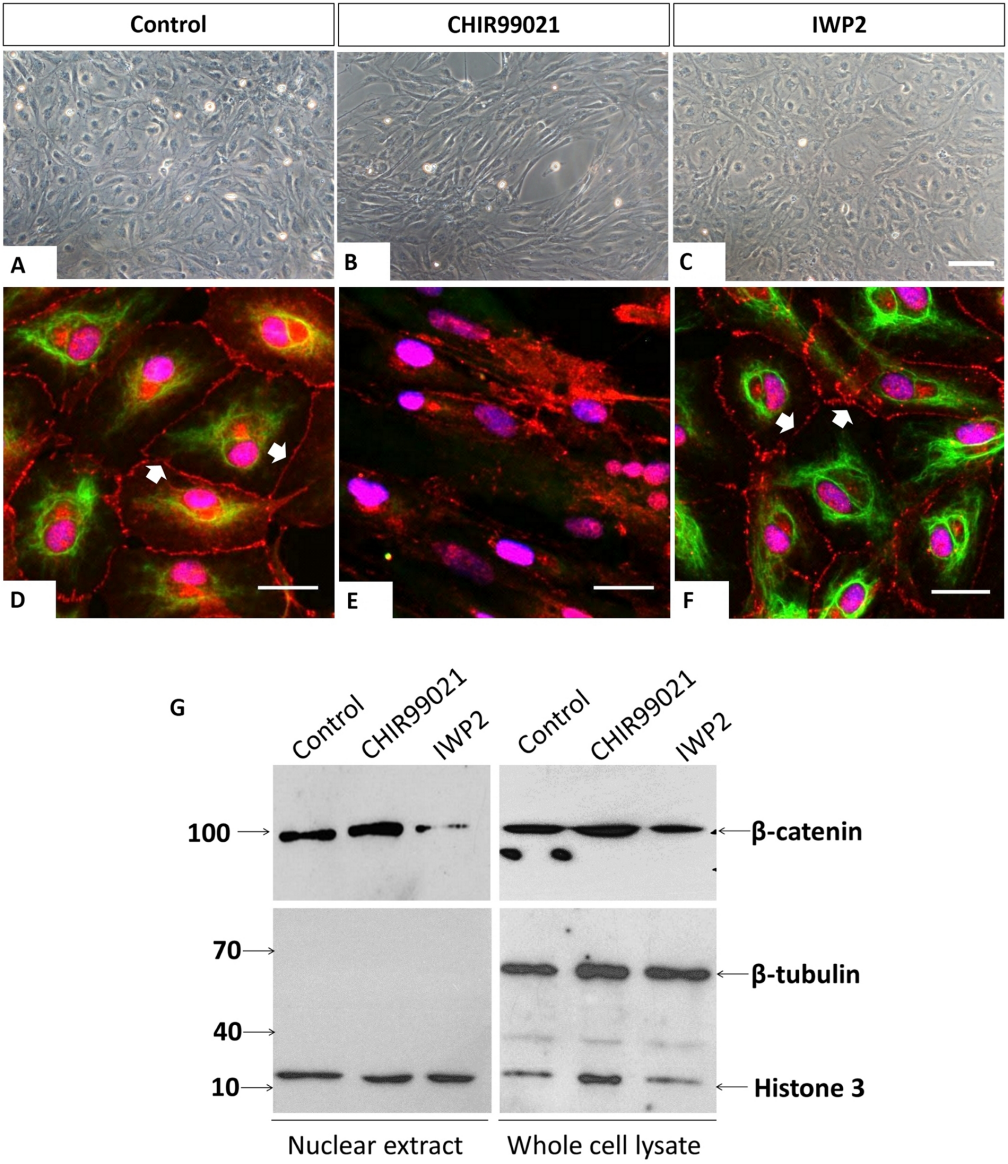
**CK regulates plasma membrane β-catenin.** (A–C) Phase-contrast images of XtiSCs after treatment with CHIR99021 and IWP2 for 3 days show the morphological changes in CHIR99021-treated cells. XtiSCs were collected for immunofluorescence (D–F) or immunoblotting analysis (G). Immunostaining of CHIR99021-treated XtiSCs against CK (green) and β-catenin (red) reveals the disruption of the CK network and cell-to-cell contact altogether with the disappearance of membrane β-catenin. Nuclei were stained with DAPI (blue). Scale bars: 20 μm. Arrows indicate membrane-bound β-catenin. The downregulation of nuclear β-catenin in media supplemented with IWP2 was confirmed by immunoblotting (G). Histone H3 is a marker of nuclei. The absence of β-tubulin shows the purity of the nuclear lysate.

Western blot analysis of nuclear fraction revealed slightly higher accumulation of β-catenin in cell nuclei after a 3-day treatment with 3 μM CHIR99021, while no significant difference in β-catenin level of control and CHIR99021-treated cells from whole-cell lysate was observed (Fig. 7G). Cells cultivated in 2 μM IWP2 showed substantial degradation of β-catenin in the nuclear fraction (Fig. 7G). Expression level and distribution of CK and β-catenin were visualized by immunocytochemistry as well (Fig. 7D–F). We observed total loss of membrane β-catenin in cells treated with CHIR99021 compared to control. In agreement with the immunoblotting result, the downregulation of nuclear β-catenin was detected only in XtiSC incubated with IWP2, but not with CHIR99021. Notably, the expression of CK was accompanied by the presence of membrane β-catenin in vehicle control and XtiSCs cultured with IWP2 (Fig. 7D,F). This result supports the positive role of CK in the retention of β-catenin in the plasma membrane, hence the stabilization of β-catenin-based junction in immature SCs. CHIR99021 also induced morphological changes in XtiSCs from cobblestone to long-rod shape associated with the breakdown of cell-to-cell contacts (Fig. 7B,E).

**Fig. 8.**
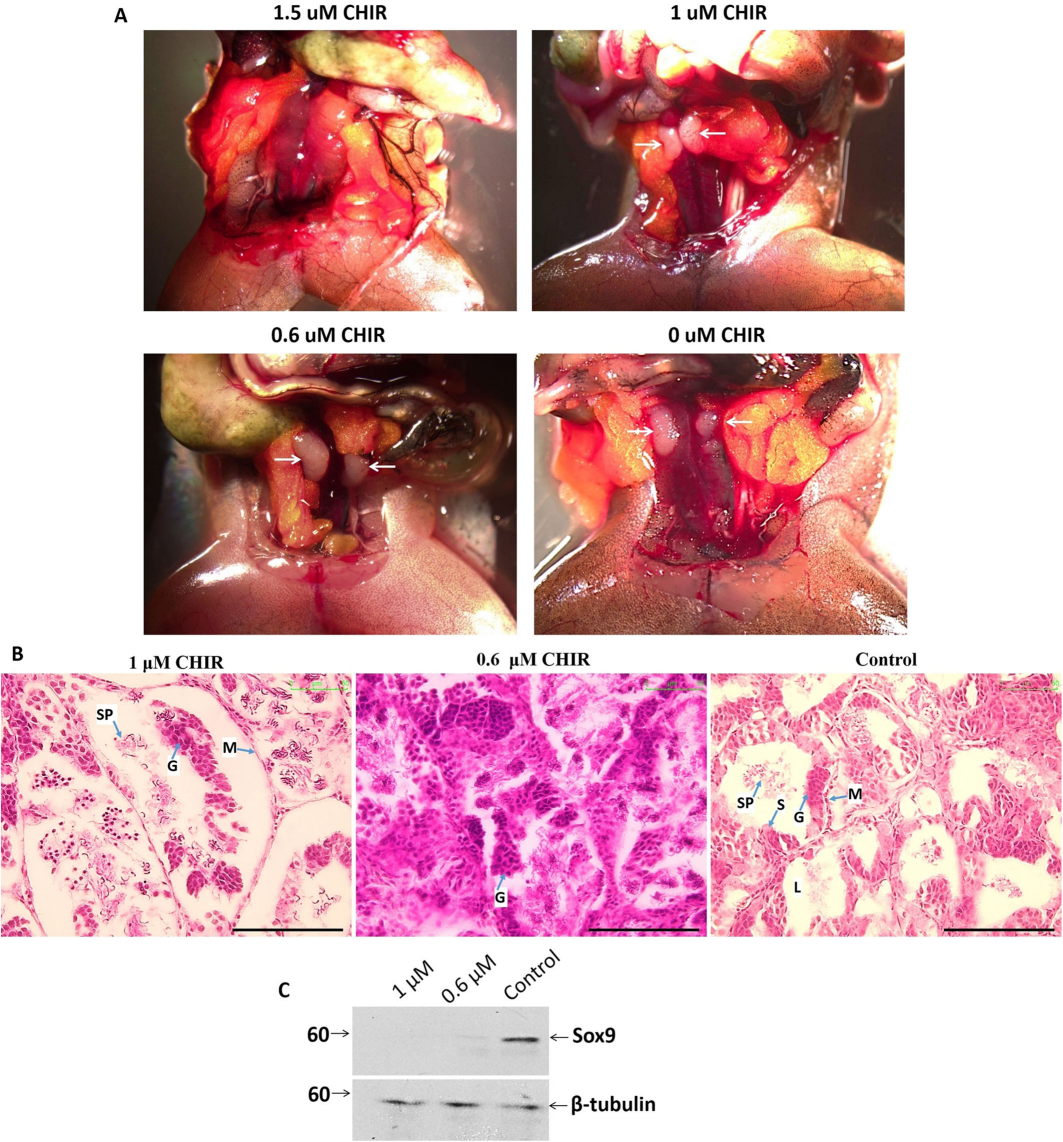
***In vivo* treatment of CHIR99021 led to failure in testicular development.** (A) 2.5 months after CHIR99021 was injected into the dorsal sac, no testes were observed in 1.5 μM-injected frogs. Arrows indicate testes. (B) H&E staining of testicular sections from 1, 0.6 and 0 μM CHIR-injected *X. tropicalis* frogs. The most serious damage, the detachment of germinal epithelium from tubules, was observed in the 1 μM group. Testes with 0.6 μM CHIR had unorganized seminiferous tubules. Scale bars: 100 μm. L, lumen; S, Sertoli cells; G, germ cells; SP, spermatid; M, mesenchyme. (C) Immunoblotting of testicular extract from 1, 0.6 and 0 μM CHIR-injected *X. tropicalis* with antibody against Sox9 (SC marker). β-tubulin is as a loading control.

To examine the role of cell junctions in SCs and testicular development, we injected 0, 0.6, 1 and 1.5 μM CHIR99021 into the dorsal sacs of 5-month-old male frogs. 2.5 months after injection, all frogs survived normally. However, no testes were found in individuals injected with 1.5 μM CHIR99021 (Fig. 8A). The testicles obtained from other experimental groups were embedded into paraffin for immunohistochemistry. H&E staining of testicular sections showed impaired morphology and structure of seminiferous tubules in CHIR99021-treated animals. The most serious damage was observed at 1 μM drug concentration, when germinal epithelium extruded away from the basement membrane and the outer myoid layer (Fig. 8B). Lower concentration of CHIR99021 (0.6 μM) resulted in unorganized-seminiferous tubules (Fig. 8B). In comparison with vehicle control individuals, Sox9 protein, a specific maker of SCs, was significantly reduced in testes derived from injected frogs as visualized by western blot analysis (Fig. 8C). Consequently, only a few sperm cells were found in testis from 1 μM CHIR99021-injected animals (Fig. 8B).

The data indicate the potential role of CK in SC development via the retention of cell-to-cell contacts mediated by the β-catenin in the adhesion complex. The suppression of CK by CHIR99021 inhibited the maturation of SCs and potentially causes the failure of testicular development.

## DISCUSSION

CKs, like other IFs, lack plus and minus ends, thus they have not been thought to be involved in a basic network for intracellular transport. However, several studies have revealed the key role of IF and CK in vesicular trafficking. Depolymerization of IF resulted in the strong inhibition of vesicle transport towards the plasma membrane in astrocytes (Potokar et al., 2007). SNARE proteins form the large protein complex, which is essential for docking and the fusion of vesicles with cytoplasmic membrane. Delivery of Syntaxin 3, an important component of SNAREs to the apical domain of plasma membrane, was impaired in CK 8-deficient mice, leading to mis-targeted trafficking of a number of apical proteins (Ameen et al., 2001). The mislocalization of GLUT-1 and -3 from the apical compartment was identified in embryonic epithelia of CK-null mice (Vijayaraj et al., 2009). Moreover, CK is essential for the retention of proteins at their proper positions. Knockdown of CK caused the Golgi enzyme, Core 2 *N*-acetylglucosaminyltransferase 2/M (C2GnT-M), to leave outside the Golgi complex followed by its ubiquitination and degradation (Petrosyan et al., 2015).

In epithelial cells, β-catenin binds to E-cadherin at the plasma membrane to form adherens junctions, which maintain cell integrity and they are involved in several intracellular signaling pathways. β-catenin can also accumulate in the nucleus and act as a key mediator of Wnt signaling. Using GFP-tagged β-catenin and a photobleaching assay exhibited high turnover rate and β-catenin transport between the nucleus and the plasma membrane in NIH 3T3 cells (Johnson et al., 2009). The balance of β-catenin activity between Wnt signaling and cell-to-cell contacts is regulated by E-cadherin (Fagotto et al., 1996; Heasman et al., 1994) via modulation of the vesicle-associated transport (Chairoungdua et al., 2010). Moreover, transfection of cultured cells with GFP-tagged CK mutant (GFP-CK18 R89C) led to the disruption of the CK network. Subsequently, several junction-associated proteins, including β-catenin, detached from the cell membrane and colocalized with CK aggregates (Hanada et al., 2005). E-cadherin was also mis-targeted in these CK mutant-transfected cells. This study shows the association between β-catenin retention at the plasma membrane and CK expression in immature SCs *in vitro* and *in vivo*. Both proteins co-expressed in juvenile testes and *in vitro* cultured immature SCs (Figs 1 and 4). The transient disruption of the CK network by acrylamide induced the breakdown of membrane-bound β-catenin in XtiSCs (Fig. 4) but not in the opposite manner. The loss of β-catenin-based adherens junctions by EGTA did not result in the suppression of the CK network (Fig. 4). The negative effect of acrylamide on the CK network could be linked to its interaction with EGF receptor, thus blocking EGF signaling (Smaill et al., 1999). EGF has been reported to regulate keratin phosphorylation, inducing the reorganization of keratin filaments (Makarova et al., 2013; Ku and Omary, 1997). Moreover, experimental observation of CK loss after the stimulation of primary kidney cells and bladder cancer organoids with CHIR99021 has been reported previously (Francipane and Lagasse, 2015; Yoshida et al., 2018). Our results are in agreement with these studies. CK was suppressed in XtiSCs (immature SCs) by CHIR99021 (Fig. 7) and in adult testes (Fig. 1). Simultaneously, the absence of membrane β-catenin was observed. CHIR9901 impaired the testicular development *in vivo* as a consequence of the dedifferentiation of immature SCs (Fig. 8). In spite of no direct evidence of CK and β-catenin binding, our experimental data suggest the capacity of CK in the regulation and the maintenance of β-catenin-based junctions in immature SCs.

Glycogen synthase kinase 3 (GSK3), a serine/threonine protein kinase, has over 100 substrates, including β-catenin and Snail1, resulting in their degradation after phosphorylation. Regarding testicular development, the downregulation of GSK3β has been reported in non-obstructive azoospermic men (Nazarian et al., 2014). The disruption of GSK3α had a negative effect on sperm motility of KO mice (Bhattacharjee et al., 2015). In this study, we showed that cultivation of immature SCs derived from testes of juvenile *X. tropicalis* males with GSK3 inhibitor, CHIR99021, could downregulate the CK expression and consequently disaggregate β-catenin-based junctions (Fig. 7). This drug was also responsible for the failure in testicular development (Fig. 8). This may indicate the role of GSK3 in the regulation of SC maturation. Interestingly, CHIR99021 did not significantly increase the accumulation of β-catenin in XtiSCs (Fig. 7B). Instead, we observed the stability of another nuclear GSK3 substrate, Snail1 (Nguyen et al., 2019), which has been reported to suppress CK expression via the transcription factor Zeb1 and activated STAT3 signaling pathway (Kaufhold and Bonavida, 2014; Wu et al., 2012). XtiSC cultivation in the medium with Wnt inhibitor IWP2 led to the upregulation of CK expression probably through the Snail1 inhibition by the reactivated GSK-3. As expected, we observed downregulation of the nuclear β-catenin, however, membrane β-catenin was not affected due to the functional CK network (Fig. 7F).

XtiSCs represent a unique cell model for the identification of key molecules in SC maturation. This study reveals a potential role of CK in maintaining immature SC junctions via the retention of plasma membrane β-catenin, contributing to proper testicular development and spermatogenesis.

## MATERIALS AND METHODS

All materials were purchased from Sigma-Aldrich, except where noted.

### Ethical statement

This study was carried out in strict accordance with the Act No. 246/1992 Coll. on the protection of animals against cruelty. Official permission was issued to the Faculty of Science, Charles University in Prague by the Ministry of Education, Youth and Sports of the Czech Republic, No. MSMT-37376/2014-4.

### Histochemistry and immunohistochemistry staining

*Xenopus tropicalis* testes were collected at indicated time points, fixed overnight in MEMFA (0.1 M MOPS, 2 mM EGTA, 1 mM MgSO4 and 3.7% formaldehyde) at 4°C, rinsed, dehydrated and embedded into paraffin for sectioning by microtome. 8 μm testicular sections in the middle of testis were then rinsed in xylene and rehydrated in ascending ethanol gradient. Several sections were stained by H&E following the standard protocol.

For immunofluorescence staining, testes were collected and prepared for vibratome sectioning as described elsewhere (Tlapakova et al., 2016). If antibody against E-cadherin was used, testes were fixed in freshly prepared Dent's fixative (methanol:DMSO, 4:1) instead of MEMFA overnight at 4°C. 30 μm agarose-embedded sections were permeabilized with 0.2% Triton X-100 for 1 h and incubated with primary antibodies, including CK (2 μg/ml, 1h5, DSHB), β-Catenin (1:500, C2206) and E-cadherin (1:150, 33,4000, Invitrogen) for 3 days at 4°C. Sections were rinsed thoroughly in PBS overnight at 4°C before immersing into secondary antibodies conjugated with Alexa Fluor 488 anti-mouse or Alexa Fluor 594 anti-rabbit (1:500, Thermo Fisher Scientific) for 4 h at room temperature (RT). Cell nuclei were visualized by DAPI. Sections were stained with secondary antibodies only used as a negative control.

### XtiSC culture and fluorescent immunostaining

XtiSCs were obtained and cultured as previously described (Tlapakova et al., 2016). Cells proliferated in growth medium overnight and then in the induction medium supplemented with acrylamide (10 mM) for 75 min, or EGTA (2 mM) for 15 min, or LiCl (10 - 40 mM) for 24 h, or CHIR99021 (CHIR) (3 μM) or IWP2 (2 μM) for 3 days. The CHIR99021 and IWP2 stock solutions were prepared in DMSO according to the manufacturer's instructions. The concentration of DMSO in culture medium was kept lower than 0.1%. The same concentration of DMSO was added in the culture medium of vehicle control.

For immunofluorescence staining, a 20 μl drop of 10,000 cells was plated in the center of 12-mm diameter coverslip glasses coated with 0.01% poly-L Lysine or collagen type I (2.5 μg/cm^2^). The growth medium was added after cells attached to the glass surfaces and was exchanged with indicated medium after 24 h. Cells were collected at indicated times, fixed with 2% formaldehyde for 20 min at RT or in Dent's fixative overnight at 4°C (for antibody against Sox9), then permeabilized with 0.1% Triton X-100 for 15 min and incubated with primary antibodies, including CK type II (2 μg/ml, mouse, 1h5, DSHB), β-catenin (1:500, rabbit, C2206), Sox9 (1:300, rabbit, HPA001758), focal adhesion kinase (FAK) (1:1000, rabbit, F2918), β-tubulin (1:200, mouse, T4026), integrin β1(2 μg/ml, mouse, 8C8, DSHB), followed by secondary antibodies conjugated with Alexa Fluor 488 anti-mouse or Alexa Fluor 594 anti-rabbit (1:500, Thermo Fisher Scientific). F-actin was visualized by Alexa Fluor 647-conjugated phalloidin (1:100, A22287, Thermo Fisher Scientific) for 1 h at RT. DAPI was used for cell nuclei staining. Cells were stained, with secondary antibodies only used as a negative control.

### Karyotype analysis

0.02 μg/ml colchicine was added into the culture medium with XtiSCs at the exponential growth phase for 4.5 h to arrest cells at metaphase. Cells were then harvested by trypsin, gently dispersed in amphibian hypotonic solution (0.038 M KCl, 1.9 mM HEPES, 0.038 mM EGTA, 1% Triton X-100, pH 7.3), followed by the fixation in Carnoy's fixative (methanol:acetic acid ratio 3:1) for 15 min. The fresh fixative was replaced three times. The cell suspension was dropped onto a clean microscopic slide to spread out metaphase chromosomes. Just before drying out, slides were immersed into 50% acetic acid to remove cell membranes and cytoplasm traces. Slides were then stained with Giemsa and observed under a bright field microscope. A minimum of 15 G-banded metaphase chromosomes were analyzed.

### Soft agar colony formation assay

The soft agar colony formation assay allows *in vitro* evaluation of anchorage-independent growth, one of the hallmarks of malignant cell transformation. XtiSCs at passage 5 and 12 were harvested by papain to get a single cell suspension, counted using cell counter (Thermo Fisher Scientific), and resuspended in growth medium. 24,000 harvested cells/well of a six-well plate were mixed with medium containing 0.3% agar, and overlaid on the semi-medium containing 0.6% agar in triplicates. The plates were replenished with fresh medium at an interval of 2 or 3 days. Colonies were visualized under a stereomicroscope after 3 weeks. Simultaneously cultured HeLa cells served as a positive control. Colonies greater than 100 microns were considered as transformed. Three independent assays were done.

### Immunoblotting

Cells were cultured in indicated medium for 3 days before nuclear extraction by the amphibian hypotonic solution (0.038 M KCl, 1.9 mM HEPES, 0.038 mM EGTA, 1% Triton X-100, pH 7.3) and vortexed. The homogenate was centrifuged to separate the nuclear fraction (pellet) from the supernatant (cytoplasmic fraction). For the subsequent immunoblotting analysis the nuclear pellets or whole cells or testes were lysed by RIPA lysis buffer containing cocktail of protease inhibitors (1:100, Promega) following the standard protocol as described (Towbin et al., 1992). Both samples were then dissolved in sample buffer for SDS-PAGE and sonicated using a SONIFER 250 (Branson), followed by boiling for 5 min. 10 μg of total proteins were separated by SDS-PAGE in 10% polyacrylamide gel. After electrophoresis, proteins were transferred onto nitrocellulose membrane and incubated with ﬁrst antibody, including antibodies against β-tubulin (1:2000, T8328), β-catenin (1:5000, C2206), histone H3 (1:1000, ab18521, Abcam) and Sox9 (0.4 μg/ml, HPA001758). Bound antibodies were then visualized using horseradish peroxidase-conjugated secondary antibody followed by acridan-based chemiluminescent HRP substrate for detection using X-ray film (32132, Thermo Fisher Scientific).

### *In vitro* fertilization and *in vivo* CHIR99021 treatment

*Xenopus tropicalis* embryos were produced and cultivated by the standard *in vitro* fertilization procedure (Geach and Zimmerman, 2011). Young frogs (three for each experimental group), 4.5–5 months after metamorphosis, were injected with 20 μl of CHIR99021 at 0.6, 1, 1.5 μM (in 7.5% DMSO and 92.5% saline) or vehicle (7.5% DMSO and 92.5% saline) into the dorsal lymphatic sac daily for 4 days and monitored 2.5 months after treatment as described in mouse and zebrafish (Chen et al., 2014; Pachenari et al., 2017).

### Origin of experimental animals and breeding

The *X. tropicalis* animals, Ivory Coast strain, were purchased from University of Portsmouth and bred in 50 l tanks in tap water filtered through the active carbon filter and with added sea salt up to the conductivity of 1200 μOsm. Juvenile males were sacrificed at the age of 5 months and adults at 3 years using 0.4% MS222 (Sigma-Aldrich) and 0.4% NaHCO3 anesthetic solution and then were decapitated with scissors. The testicles were isolated from experimental frogs at indicated time points and sectioned for immunohistochemistry.

### Statistics

All assays were repeated in three independent experiments.
